# Clinical effects of *Bifidobacterium Longum Subsp. Infantis* YLGB-1496 on children with respiratory symptoms

**DOI:** 10.3389/fnut.2025.1537610

**Published:** 2025-02-19

**Authors:** Pin Li, Uma Mageswary, Adli Ali, Fahisham Taib, Thai Hau Koo, Azianey Yusof, Hua Jiang, Hanglian Lan, Weilian Hung, Min-Tze Liong, Yumei Zhang

**Affiliations:** ^1^Department of Nutrition and Food Hygiene, School of Public Health, Peking University Health Science Center, Beijing, China; ^2^School of Industrial Technology, Universiti Sains Malaysia, Penang, Malaysia; ^3^Department of Pediatrics, UKM Medical Centre, Faculty of Medicine, Universiti Kebangsaan Malaysia, Kuala Lumpur, Malaysia; ^4^Pediatric & Palliative Care, Hospital Universiti Sains Malaysia, Kubang Kerian, Kelantan, Malaysia; ^5^Kepala Batas Health Clinic, Penang, Malaysia; ^6^School of Nursing, Peking University, Beijing, China; ^7^National Center of Technology Innovation for Dairy, Hohhot, China; ^8^Inner Mongolia Dairy Technology Research Institute Co., Ltd., Hohhot, China

**Keywords:** *Bifidobacterium longum subsp. infantis* YLGB-1496, respiratory symptoms, immunological effects, toddlers, randomized controlled trial

## Abstract

**Objectives:**

This study aimed to examine the effects of *Bifidobacterium longum subsp. infantis* YLGB-1496 (*B. infantis* YLGB-1496) on the frequency of respiratory illness symptoms and immunity profiles among toddlers.

**Methods:**

In this double-blind, randomized, placebo-controlled, 12-week intervention study, toddlers with at least 2 respiratory illness symptoms were randomly assigned into the probiotic (YLGB-1496) or placebo group at a 1:1 ratio. Follow-up examinations were conducted at baseline (week 0) and at weeks 6 and 12 of the intervention. The frequency of respiratory illness symptoms was assessed at these time points using validated questionnaires. Oral swabs and fecal samples were collected from participants at weeks 0, 6, and 12 to examine inflammatory cytokines.

**Results:**

Among the 120 toddlers initially included in the study, 115 completed the 12-week intervention (58 in the YLGB-1496 group and 57 in the placebo group). The risk of antibiotic use or clinical visits was significantly lower in the YLGB-1496 group than in the placebo group (antibiotic use odds ratio (OR) = 0.37 [0.369, 0.372]; clinical visit, OR = 0.743 [0.741, 0.744]), but these differences were nonsignificant after adjusting for other potential confounders (*p* > 0.05). The YLGB-1496 group presented a lower incidence of several respiratory symptoms than the placebo group, including fever (*p* < 0.001), cough (*p* < 0.001), sneezing (*p* = 0.012), nose block (*p* = 0.001), and runny nose (*p* < 0.001). The results also revealed that the salivary cortisol concentration was significantly lower in the YLGB-1496 group than in the placebo group (*p* = 0.026), but no effects on INF-*γ*, IL-1β, IL-13, IL-4, or IL-10 were detected.

**Conclusion:**

*Bifidobacterium infantis* YLGB-1496 may serve as a potential natural, nonpharmacological strategy for the safe management of respiratory tract issues in toddlers.

**Clinical trial registration:**

The trial was registered at ClinicalTrials.gov (identifier number NCT05794815).

## 1 Introduction

Probiotics are active microorganisms that colonize the human body and confer various benefits to the host. This concept was first proposed by Stillwell R. H. and Lilly D. M. in 1965 (1). The Food and Agriculture Organization of the United Nations, in conjunction with the World Health Organization, defines probiotics as living microorganisms that provide health benefits to their host when they are administered in adequate quantities (2). The most widely recognized probiotics include various strains of Lactobacillus and Bifidobacterium. Notably, *Bifidobacterium longum subsp. infantis* (*B. infantis*) may play a crucial role during early life stages. Previous studies have demonstrated that this specific subspecies enhances immune function in infants and toddlers (3–5).

Respiratory diseases represent a significant global health issue, especially among infants and toddlers (6). Common respiratory symptoms in these age groups include fever, chills, sore throat, headache, cough, and runny nose. Children who experience respiratory tract infections usually have immunity responses such as high levels of changes in cytokines signal inflammation and intestinal barrier disruption due to invasions of pathogens. The use of antibiotics has been proven to be effective in treating respiratory infections, but their overuse is a widespread concern among affected individuals (7). Previous studies have shown that supplementing Bifidobacterium probiotics early in life can effectively reduce the frequency of respiratory symptoms (8, 9).

The gut serves as the largest immune organ within the body. In the gut, stimulation from food, environmental antigens, and the intestinal flora postnatally is essential for developing an effective intestinal immune system (10). The surface components of *B. infantis*, such as S-protein, lipoteichoic acid (LTA), and exopolysaccharides (EPS), exhibit stimulatory effects similar to those induced by pathogenic microorganisms (11). In addition to contributing to biological barriers, these components influence immune barriers (intestinal-associated lymphoid tissue and secretory antibodies), thereby modulating intestinal immunity.

*Bifidobacterium longum subsp. infantis* YLGB-1496 (*B. infantis* YLGB-1496) is isolated from human breast milk. It is able to maintain a high survival rate in acidic or bile salts mimics artificial gastrointestinal environments, and it strongly adheres to Caco-2 cells derived from the human intestinal epithelium. Furthermore, this probiotic has shown significant immunoregulatory capabilities (12).

However, most relevant studies were cell-based or animal studies, and there is a paucity of data regarding human subjects among toddlers. Consequently, this study aimed primarily to assess differences in the frequency of respiratory illness symptoms between toddlers receiving *B. infantis* YLGB-1496 and those receiving placebo as well as to evaluate variations in immunity profiles between these two groups.

## 2 Materials and methods

### 2.1 Subjects

Subjects were recruited from the Universiti Kebangsaan Malaysia (UKM) campus as well as from the community. The inclusion criteria were as follows: (a) aged 1–3 years, and (b) exhibiting at least two of the following signs and symptoms: fever, nasal obstruction, chills, sore throat, headache, cough, runny nose, olfactory disturbances, or taste disturbances. The exclusion criteria were as follows: (a) taking long-term medication (>6 months) for any disease; (b) any deformities or metabolic/chronic diseases; (c) conditions or interventions that may interfere with the study(judged by professional pediatricians); (d) consumption of nutritional supplements containing probiotics within 2 weeks before the intervention; (e) intake of foods for special medical purposes or nonstandard formula powders due to lactose intolerance and galactosemia; and (f) participation in other clinical studies within 4 weeks before the intervention. Informed consent was obtained from the parents or legal guardians of the children before the commencement of the study.

### 2.2 Products

Both probiotic and placebo products (2 g/bag) were manufactured under HACCP conditions and were stored at a temperature below 4°C to ensure that the probiotics remained active as much as possible. According to previous researches, a high dose of *B. infantis* (1.8–2.8*10^10^ CFU/day) was safe and well-tolerated in healthy breastfed and/or formula-fed infants (13, 14). The probiotic product contains about 1.5*10^10^ colony-forming units (CFU)/g viable *B. infantis* YLGB-1496 during production. After bagging, transportation, and storage, three batches of placebo products are randomly selected for viable bacteria counting. After storage for 3–6 months, each probiotic product contains about 1*10^10^CFU *B. infantis* YLGB-1496.

### 2.3 Methods

In this double-blind, randomized, placebo-controlled, 12-week intervention study, qualified subjects were randomly assigned into the probiotic (YLGB-1496) or placebo group at a 1:1 ratio. Randomization was performed by the study statistician, who had no contact with the participants, using a computer-generated process in Microsoft Excel (version 2016) to ensure unbiased allocation of participants to experimental groups. The allocation sequence was not revealed to any research team member until the study’s completion. This study was conducted in accordance with the Declaration of Helsinki, where all procedures involving human subjects were approved by the UKM Research Ethics Committee (No. UKM/PPI/111/8/JEP-2023-074) and the Medical Ethics Research Board of Peking University (No. IRB00001052-22166). The trial was registered at ClinicalTrials.gov (identifier number NCT05794815).

The probiotic group received one sachet(2 g/bag) of the probiotic product only contains *B. infantis* YLGB-1496 and maltodextrin per day in plain water or milk at a temperature not exceeding 40°C. The placebo group received one sachet of placebo (maltodextrin, 2 g/bag) per day in the same package. The intervention probiotic powder and placebo had the same outer packaging and were only distinguished by different production lot numbers. The grouping codes were revealed once the statistical analysis was completed. Follow-up surveys were conducted at baseline (week 0) and at weeks 6 and 12 of the intervention.

### 2.4 Sample size

The sample size was calculated for a parallel group study design based on power design analysis. Previous studies have shown that for an intervention in which probiotics are used to reduce clinical visits in children for respiratory diseases, a standard deviation of 0.46 times within the group was observed, accompanied by a 0.32-fold reduction between the treatment and placebo groups (15). This calculation was based on the need for a continuous response variable from independent control and experimental subjects, with a ratio of power to subject fixed at 1:1, a probability of 0.95, and a type-I error probability associated with this test of the null hypothesis of 0.05. Considering a potential dropout rate of 10%, each group required a sample size of 60, and a total of 120 subjects was needed for this study.

### 2.5 Data collection

#### 2.5.1 Questionnaires

Eligible subjects who met all the inclusion and exclusion criteria were asked to complete two types of questionnaires: (a) a demographic questionnaire (collected at baseline week 0) and (b) a respiratory health questionnaire (collected at weeks 0, 6, and 12). All the questionnaires were validated and translated into the Malay language (16). The short Food Frequency Questionnaire was used to investigate the children’s dietary status during the intervention, which mainly assessed the consumption rates of various foods and nutritional supplements. Adverse events (AEs) and serious adverse events (sAEs) that occurred during the trial were recorded, and their correlation with the tested products was evaluated by doctors.

#### 2.5.2 Sample collection and immunological analysis

At weeks 0, 6, and 12, oral swab samples were collected from each toddler from the inside of both the left and right cheeks, one immediately after the other. At the same time points, fecal samples were also collected in fecal collection tubes and glass beads by the subjects. All the tubes were capped tightly and stored at −80°C until further analyses. These samples were collected by our medical team.

The fecal samples were analyzed for the concentrations of cytokines, including inter-feron-*γ* (IFN-γ), interleukin-1β (IL-1β), IL-4, IL-10, and IL-13. The oral swab samples were collected for the analysis of cortisol. All the biomarkers were tested via commercial enzyme-linked immunosorbent assay (ELISA) kits in accordance with the manufacturer’s instructions (Sunlong Biotech, Hangzhou, China).

### 2.6 Statistical analysis

Intention-to-treat analysis was performed via R software version 4.2.3. For basic information, the data are presented as the mean value ± standard error unless otherwise stated. Differences in continuous variables between groups were compared via Student’s t-test, whereas categorical variables were compared via the chi-square test. Poisson regression was used to analyze the effect of the intervention on the incidence of respiratory symptoms. A linear or generalized linear mixed model was constructed to analyze the impact of the intervention on the outcomes. All tests were two-sided, with *p* < 0.05 considered to indicate statistical significance.

## 3 Results

### 3.1 Sociodemographic characteristics

**Figure 1:**
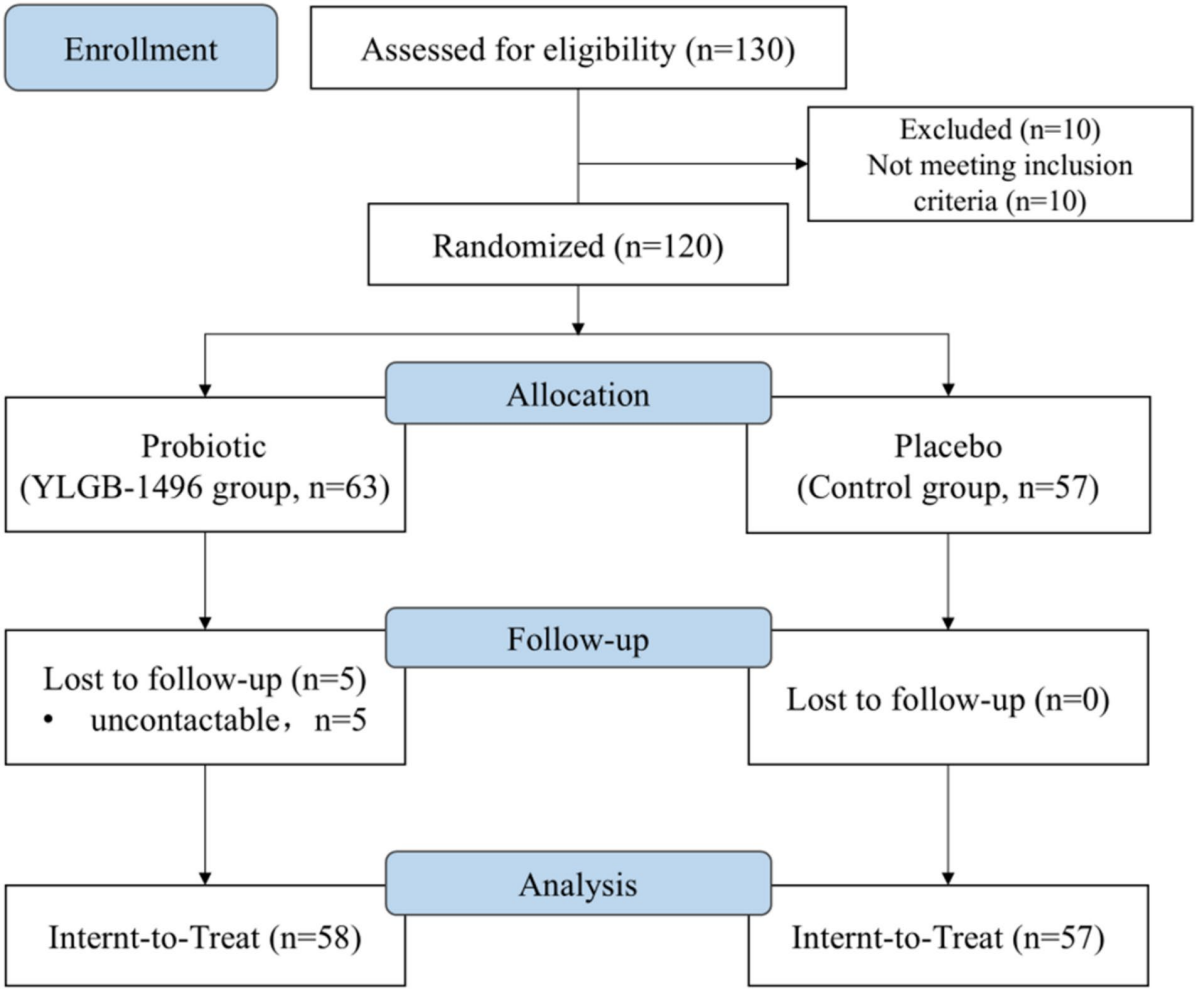
CONSORT flow diagram of the study protocol.

A total of 130 volunteers were recruited for the study, and 120 met the inclusion and exclusion criteria and signed the informed consent form after the initial screening. A total of 115 people ultimately completed the study (58 in the experimental group and 57 in the placebo group; Figure 1). All 115 subjects provided oral samples and answers to the questionnaires. No adverse effects were reported throughout the study, and no subjects dropped out due to any complications. The sociodemographic characteristics of the participants in the different groups are summarized in Table 1. The proportion of children who were delivered by cesarean section and who were mainly cared for by family was greater in the placebo group than in the YLGB-1496 group. There was no significant difference in other sociological demographic factors between the two groups.

**Table 1:** Demographic characteristics of the participants at enrollment.

Variable	YLGB-1496 (*n* = 58)	Placebo (*n* = 57)	*p* value
Age (mean ± SD, months)	29.88 ± 8.44	30.58 ± 7.98	0.649^a^
Sex (n/%)			0.515^b^
Female	32 (55.17)	27 (47.37)	
Male	26 (44.83)	30 (52.63)	
Delivery method (n/%)			**0.049**^b^
Cesarean section	14 (24.14)	5 (8.77)	
Vaginal delivery	44 (75.86)	52 (91.23)	
Gestational weeks (n/%)			0.371^b^
<37	4 (6.90)	1 (1.75)	
≥37	54 (93.10)	56 (98.25)	
Primary caregiver (n/%)			**0.003**^b^
Family care	34 (58.62)	43 (75.44)	
Daycare center	3 (5.17)	8 (14.04)	
Family and daycare center	21 (36.21)	6 (10.53)	
Feeding patterns (n/%)			0.785^b^
Exclusive breastfeeding	4 (6.90)	6 (10.53)	
Mixed feeding	12 (20.69)	11 (19.30)	
Artificial feeding	42 (72.41)	40 (70.18)	
Duration of breastfeeding (n/%)			0.436^b^
≤6 m	41 (70.69)	41 (71.93)	
6–12 m	3 (5.17)	6 (10.53)	
>12 m	14 (24.14)	10 (17.54)	
Household size (n/%)			0.779^b^
≤4	30 (51.72)	27 (47.37)	
>4	28 (48.28)	30 (52.63)	
House location (n/%)			0.529^b^
Urban	37 (63.79)	35 (61.40)	
Suburban	13 (22.41)	17 (29.82)	
Rural area	8 (13.79)	5 (8.77)	
Family income (RM, n/%)			0.940^b^
<3,000	16 (27.59)	14 (24.56)	
3,000–6,000	24 (41.38)	24 (42.11)	
6,000–10,000	10 (17.24)	9 (15.79)	
>10,000	8 (13.79)	10 (17.54)	
History of food allergy (n/%)			1.000^b^
TRUE	3 (5.17)	2 (3.51)	
FALSE	55 (94.83)	55 (96.49)	
Having pets at home (n/%)			0.637^b^
TRUE	15 (25.86)	18 (31.58)	
FALSE	43 (74.14)	39 (68.42)	

Bold text represents *p* < 0.05.

a *p value obtained via Student’s t test.*

b *p value obtained via the chi-square test.*

### 3.2 Food and nutritional supplements consumption

The consumption rates of various food and nutritional supplements during the intervention are shown in Supplementary Table S1. At baseline, the use of breast milk was significantly greater in the YLGB-1496 group than in the placebo group, and the consumption rate of vegetables in the YLGB-1496 group was lower at baseline and 12 weeks. The consumption rates of other food types and nutrient supplements were not significantly different between the two groups and did not change over time during the intervention period (*p* > 0.05).

### 3.3 Antibiotic use and clinical visits due to respiratory illness symptoms during the intervention

A generalized linear mixed model was used to analyze the risk of antibiotic use and clinical visits due to respiratory illnesses during the intervention between groups. In Model 1, which was adjusted for only baseline values, time, group, and interaction terms com-prising time and group, the risk of antibiotic use or clinical visit in the YLGB-1496 group was significantly lower than that in the placebo group (antibiotic use odds ratio, OR = 0.37 [0.369, 0.372]; clinical visit odds ratio, OR = 0.743 [0.741, 0.744]), but there was no statistically significant difference between the two groups after further adjustment for other possible confounders (Table 2).

**Table 2:** Antibiotic use and clinical visits due to respiratory illnesses during the intervention (n/%).

Variable	week	YLGB-1496	Placebo	*p* value^a^	*p* value for intervention^b^		
					Model 1	Model 2	Model 3
Antibiotic use	0	8 (13.79)	5 (8.77)	0.578	**<0.001**	0.786	0.201
	6	5 (8.62)	6 (10.53)	0.976			
	12	6 (10.34)	5 (8.77)	1.000			
Clinical visit	0	13 (22.41)	9 (15.79)	0.505			
	6	8 (13.79)	8 (14.04)	1.000	**<0.001**	0.902	0.264
	12	9 (15.52)	9 (15.79)	1.000			

Bold text represents *p* < 0.05.

a *p value obtained via the chi-square test.*

b *p values for the intervention were obtained from the generalized linear mixed model.*

Model 1: adjusted for baseline values, time, group, and interaction terms comprising time and group. Model 2: additionally adjusted for delivery and primary caregiver to correct for differences in the baseline characteristics of the children enrolled in the trial. Model 3: additionally adjusted for breastmilk and vegetable intake during the intervention.

### 3.4 Respiratory health

After further adjustment for possible confounders, the YLGB-1496 group presented a lower incidence of several respiratory symptoms than the placebo group (Table 3), including fever, cough, sneezing, nose block, and runny nose. Although other symptoms were not significantly different between the two groups according to the generalized linear mixed model, the incidence rate ratio (IRR) also showed a decreasing trend during the intervention. Despite having a greater incidence of symptoms of wheezing, poor appetite, and vomiting at baseline (week 0) than the placebo group did, the YLGB-1496 group eventually had a significantly lower incidence rate than the placebo group did at week 6 or week 12.

**Table 3:** Incidence of respiratory illness symptoms during the intervention.

Variable	Week	YLGB-1496		Placebo		IRR(95% CI)	*p* value^a^	*p* value for intervention^b^		
		Events	IR(SE)	Events	IR(SE)			Model 1	Model 2	Model 3
Fever	0	216	3.72(0.76)	266	4.67(0.60)	0.80(0.67,0.95)	**0.014**	**<0.001**	**<0.001**	**<0.001**
	6	100	1.72(0.68)	280	4.91(0.67)	0.35(0.28,0.44)	**<0.001**			
	12	84	1.45(0.63)	264	4.63(0.64)	0.31(0.24,0.40)	**<0.001**			
Cough	0	696	12.00(1.50)	580	10.18(1.10)	1.18(1.06,1.32)	**0.003**	**<0.001**	**<0.001**	**<0.001**
	6	202	3.48(0.95)	608	10.67(1.07)	0.33(0.28,0.38)	**<0.001**			
	12	184	3.17(0.95)	536	9.40(1.08)	0.34(0.28,0.40)	**<0.001**			
Sneezing	0	418	7.21(0.95)	290	5.09(0.58)	1.42(1.22,1.65)	**<0.001**	**0.001**	**0.004**	**0.012**
	6	188	3.24(0.88)	296	5.19(0.57)	0.62(0.52,0.75)	**<0.001**			
	12	106	1.83(0.57)	290	5.09(0.58)	0.36(0.29,0.45)	**<0.001**			
Nose block	0	638	11(1.49)	448	7.86(1.13)	1.40(1.24,1.58)	**<0.001**	**<0.001**	**0.001**	**0.001**
	6	190	3.28(0.95)	500	8.77(1.20)	0.37(0.32,0.44)	**<0.001**			
	12	106	1.83(0.68)	466	8.18(1.16)	0.22(0.18,0.27)	**<0.001**			
Wheezing	0	116	2(0.61)	86	1.51(0.47)	1.33(1.00,1.76)	**0.048**	0.482	0.641	0.198
	6	96	1.66(0.52)	94	1.65(0.42)	1.00(0.75,1.33)	0.980			
	12	66	1.14(0.49)	98	1.72(0.48)	0.66(0.48,0.90)	**0.010**			
Sore throat	0	106	1.83(0.43)	96	1.68(0.65)	1.09(0.82,1.43)	0.562	0.688	0.856	0.683
	6	74	1.28(0.45)	86	1.51(0.47)	0.85(0.62,1.15)	0.290			
	12	66	1.14(0.49)	74	1.30(0.46)	0.88(0.63,1.22)	0.436			
Runny nose	0	708	12.21(1.48)	968	16.98(1.56)	0.72(0.65,0.79)	**<0.001**	**<0.001**	**<0.001**	**<0.001**
	6	208	3.59(0.88)	960	16.84(1.55)	0.21(0.18,0.25)	**<0.001**			
	12	172	2.97(0.88)	926	16.25(1.58)	0.18(0.15,0.21)	**<0.001**			
Poor appetite	0	302	5.21(0.93)	120	2.11(0.50)	2.47(2.01,3.07)	**<0.001**	**<0.001**	0.097	0.105
	6	124	2.14(0.69)	138	2.42(0.55)	0.88(0.69,1.13)	0.315			
	12	86	1.48(0.59)	126	2.21(0.55)	0.67(0.51,0.88)	**0.004**			
Hoarseness	0	100	1.72(0.42)	98	1.72(0.61)	1.00(0.76,1.33)	0.984	0.496	0.572	0.267
	6	84	1.45(0.51)	98	1.72(0.48)	0.84(0.63,1.13)	0.249			
	12	68	1.17(0.58)	70	1.23(0.39)	0.95(0.68,1.33)	0.785			
Body ache	0	88	1.52(0.41)	76	1.33(0.55)	1.14(0.84,1.55)	0.409	0.720	0.758	0.438
	6	48	0.83(0.27)	66	1.16(0.32)	0.71(0.49,1.03)	0.077			
	12	50	0.86(0.42)	54	0.95(0.29)	0.91(0.62,1.34)	0.631			
Fatigue	0	180	3.10(0.76)	142	2.49(0.72)	1.25(1.00,1.55)	0.050	0.387	0.386	0.387
	6	62	1.07(0.37)	120	2.11(0.54)	0.51(0.37,0.69)	**<0.001**			
	12	64	1.10(0.53)	108	1.89(0.54)	0.58(0.43,0.79)	**0.001**			
Vomiting	0	446	7.69(1.45)	104	1.82(0.48)	4.21(3.42,5.24)	**<0.001**	0.542	0.505	0.807
	6	60	1.03(0.3)	100	1.75(0.43)	0.59(0.43,0.81)	**0.001**			
	12	40	0.69(0.34)	94	1.65(0.42)	0.42(0.29,0.60)	**<0.001**			
Headache	0	82	1.41(0.4)	110	1.93(0.49)	0.73(0.55,0.97)	**0.033**	0.233	0.306	0.144
	6	42	0.72(0.26)	106	1.86(0.43)	0.39(0.27,0.55)	**<0.001**			
	12	24	0.41(0.2)	94	1.65(0.42)	0.25(0.16,0.39)	**<0.001**			
Thick-mucus production	0	186	3.21(0.77)	534	9.37(1.58)	0.34(0.29,0.40)	**<0.001**	0.509	0.624	0.557
	6	170	2.93(0.9)	560	9.82(1.56)	0.30(0.25,0.35)	**<0.001**			
	12	84	1.45(0.63)	400	7.02(1.25)	0.21(0.16,0.26)	**<0.001**			
Pain during swallowing	0	88	1.52(0.41)	82	1.44(0.56)	1.05(0.78,1.43)	0.729	0.620	0.660	0.210
	6	58	1.00(0.37)	72	1.26(0.33)	0.79(0.56,1.12)	0.185			
	12	50	0.86(0.42)	60	1.05(0.30)	0.82(0.56,1.19)	0.297			

Bold text represents *p* < 0.05.

IR, incidence rate; SE, standard error; IRR, incidence rate ratio; CI, confidence interval. Model 1: adjusted for baseline values, time, group, and interaction terms comprising time and group. Model 2: additionally adjusted for delivery and primary caregiver to correct for differences between groups. Model 3: additionally adjusted for breastmilk and vegetable intake during the intervention.

a *p value obtained via Poisson regression.*

b *p values for the intervention were obtained from the generalized linear mixed model.*

### 3.5 Immune-related biomarkers

Tests of cortisol in saliva samples revealed that the YLGB-1496 group had higher levels of cortisol at baseline than the placebo group. However, the cortisol concentration in the YLGB-1496 group significantly decreased at week 6 and week 12 (*p* < 0.05). After adjusting for possible confounders through the linear mixed-effects model, the salivary cortisol concentration in the YLGB-1496 group was significantly lower than that in the placebo group during the intervention period (Table 4).

**Table 4:** Cortisol and cytokine levels of the participants during the intervention (means ± SDs).

Variable	Week	YLGB-1496	Placebo	*p* value^a^	*p* value for intervention^b^		
					Model 1	Model 2	Model 3
Stress and anxiety biomarker							
Cortisol (ng/μg)	0	3.46 ± 3.75	2.30 ± 2.12	**0.044**	0.083	**0.027**	**0.026**
	6	2.94 ± 3.26	2.84 ± 2.95	0.868			
	12	1.68 ± 1.71	2.69 ± 3.51	0.055			
Proinflammatory cytokines							
INF-γ(pg/μg)	0	0.17 ± 0.09	0.27 ± 0.16	**<0.001**	0.637	0.558	0.863
	6	0.23 ± 0.15	0.25 ± 0.14	0.475			
	12	0.31 ± 0.59	0.38 ± 0.70	0.534			
IL-1β(pg/μg)	0	0.31 ± 0.12	0.36 ± 0.16	0.055	0.115	0.081	0.222
	6	0.42 ± 0.38	0.32 ± 0.19	0.083			
	12	0.42 ± 0.50	0.39 ± 0.74	0.788			
IL-13(pg/μg)	0	0.75 ± 0.37	0.85 ± 0.43	0.219	0.115	0.109	0.177
	6	1.24 ± 1.05	0.78 ± 0.59	**0.005**			
	12	1.21 ± 1.00	1.29 ± 2.36	0.794			
Anti-inflammatory cytokines							
IL-4(pg/μg)	0	0.28 ± 0.13	0.36 ± 0.21	**0.010**	0.162	0.108	0.164
	6	0.41 ± 0.33	0.34 ± 0.24	0.219			
	12	0.44 ± 0.35	0.43 ± 0.47	0.909			
IL-10(pg/μg)	0	0.37 ± 0.22	0.21 ± 0.16	**<0.001**	0.092	0.064	0.287
	6	0.48 ± 0.48	0.17 ± 0.14	**<0.001**			
	12	0.54 ± 0.85	0.27 ± 0.33	**0.029**			

Bold text represents *p* < 0.05.

SD, standard deviation; INF, interferon; IL, interleukin; Model 1: adjusted for baseline values, time, group, and interaction terms comprising time and group; Model 2: additionally adjusted for delivery and primary caregiver to correct for differences between groups; Model 3: additionally adjusted for breastmilk and vegetable intake during the intervention.

a *p value obtained via Student’s t test.*

b *p values for the intervention were obtained from the linear mixed model.*

## 4 Discussion

### 4.1 Resource identification initiative

The use of probiotics to treat or prevent respiratory diseases in children is common, and probiotics are often considered a safe nonpharmaceutical tool to enhance children’s immunity (17, 18). Probiotics may influence respiratory health in children through the gut-lung axis, with the theoretical basis rooted in their ability to modulate the gut microbiota while exerting antagonistic effects against intestinal pathogens (19). This process occurs through various mechanisms, including competition for nutrients and binding sites, acidification of the intestinal milieu, synthesis of bioactive compounds, and enhancement of both specific and non-specific immune responses (20). Probiotics have demonstrated efficacy in promoting pulmonary health across diverse age groups and environments. However, the effects of different strains on health vary, and the combination, content, supplementation method, and intake time of probiotics may also affect their health effects. Various meta-analyses have shown conflicting results, with some studies showing no significant beneficial effects on children’s respiratory diseases (21).

The various cytokines induced by probiotics are closely related to inflammatory responses. When probiotics are recognized by nonspecific immune cells, they can promote cytokine secretion or directly act on specific immune cells, further affecting cellular immunity and humoral immunity. This study analyzed the ability of *B. infantis* YLGB-1496 to relieve respiratory symptoms in children and explored its impact on salivary cortisol and fecal immunochemical compounds. The results revealed that the incidence of various respiratory symptoms (fever, cough, sneezing, nasal congestion, runny nose) in children who consumed *B. infantis* YLGB-1496 was significantly lower than that in the placebo group, and the salivary cortisol concentration was significantly lower; however, no effects on INF-*γ*, IL-1β, IL-13, IL-4, or IL-10 were detected.

The results of a study conducted by Cazzola et al. (22) in Spain revealed that the addition of *Bifidobacterium longum. Subsp. infantis* R0033 (*B. infantis* R0033) to a synbiotic preparation significantly reduced the risk of common infectious diseases. Sanctuary et al. (23) reported that after a 12-week intervention with *Bifidobacterium longum. Subsp. infantis* UCD272 (*B. infantis* UCD272), some subjects presented lower levels of the proinflammatory cytokines IL-5 and IL-13. A trial using *Bifidobacterium longum. Subsp. infantis* EVC001 (*B. infantis* EVC001) also confirmed that a 60-day intervention could significantly reduce the levels of multiple inflammatory cytokines in infant feces (24).

Type B Streptococcus infection can cause severe disease and even death in newborns. Chinese scholars compared the inhibitory effects of 42 strains of probiotics, including *B. infantis* YLGB-1496, on type B Streptococcus under heat-inactivated death conditions. The experimental results demonstrated that among the 42 heat-inactivated probiotic strains, *B. infantis* YLGB-1496 exhibited the strongest antibacterial effect (25). *B. infantis* can promote the proliferation and differentiation of T cells, weaken the response of Th2-type cells, and regulate the balance between Th1 and Th2 cells (26, 27). Some scholars compared the immunomodulatory functions of 18 strains of bifidobacteria derived from human milk and reported that *B. infantis* YLGB-1496 exhibited strong immunomodulatory capabilities, manifested as increased induction of the Th1-related cytokines IFN-*γ* and TGF-*β* and decreased production of Th2-related cytokines. This effect aids in the shift of the immune response from Th2 to Th1 polarization (12). Previous studies have confirmed that a shift toward Th2 polarization in the Th1/Th2 balance promotes the secretion of IL-5 and IL-13 (26, 28). Therefore, it is speculated that *B. infantis* YLGB-1496 has immunomodulatory effects in humans, protecting the host from inflammatory diseases.

No statistically significant differences in the risks of clinical visits and antibiotic use were detected between the two groups after adjusting for some possible confounding factors; however, the clinical visit and antibiotic use rates in the YLGB-1496 group generally showed a downward trend, whereas there was no decrease in the placebo group. Furthermore, our study considered the use of antibiotic treatment as an outcome, without further investigating the frequency or dosage of antibiotic administration. Follow-up studies with larger sample sizes and mor detailed information are still needed in the future.

The main advantage of this study is that it is the first to evaluate the effects of *B. infantis* YLGB-1496 application in children with respiratory diseases through a randomized controlled trial. This study measured changes in salivary cortisol concentrations and various anti-inflammatory and proinflammatory cytokines in feces while also considering the influence of dietary factors during data analysis. However, this study has notable limitations. First, owing to ethical considerations, no blood tests were conducted, as the participants were young. The concentration of cytokines in feces is influenced by numerous factors, which may not accurately reflect the body’s inflammatory state. Additionally, the incidence of respiratory symptoms was primarily self-reported by parents, which may have introduced reporting or recall bias into the statistical results. Finally, this study focused solely on the alleviation of adverse respiratory symptoms. Since a variety of diseases can produce similar symptoms, the findings of this study have limited applicability in clinical practice. To determine the effect and value of *B. infantis* YLGB-1496 in clinical practice, more research with larger sample sizes, different probiotic doses, and detection of blood biomarkers is still needed.

## 5 Conclusion

In conclusion, the administration of probiotics has beneficial effects on respiratory symptoms and anxiety in toddlers. Thus, the use of *B. infantis* YLGB-1496 may serve as a potential natural, nonpharmacological strategy for the safe management of respiratory tract issues in this age group. Further studies are required to explore the rational clinical utilization of *B. infantis* YLGB-1496, including the incorporation of serum-related indicators and the experimentation with varying doses of probiotics.

## Data Availability

The data that support the findings of this study are available from the corresponding author upon reasonable request.
