# Supplementary material for: Clinical effects of Bifidobacterium Longum Subsp. Infantis YLGB-1496 on children with respiratory symptoms
Source: Front Nutr. 2025 Feb 19;12:1537610. doi: 10.3389/fnut.2025.1537610 (PMC11879798; doi:10.3389/fnut.2025.1537610)
Supplement: Supplementary file 1 [file Table_1.docx]

Supplementary Material

**Supplementary Table 1.** The consumption rates of foods and nutritional supplements during the intervention (n/%).

| Variable | Week | YLGB-1496 | Control | P value | |
| --- | --- | --- | --- | --- | --- |
|  |  |  |  | group^a^ | week^b^ |
| Breastmilk | 0 | 19 (32.76) | 4 (7.02) | **0.001** | 0.272 |
|  | 6 | 10 (17.24) | 4 (7.02) | 0.164 |  |
|  | 12 | 9 (15.52) | 3 (5.26) | 0.135 |  |
| Formula | 0 | 46 (79.31) | 35 (61.40) | 0.057 | 1.000 |
|  | 6 | 37 (63.79) | 35 (61.40) | 0.943 |  |
|  | 12 | 32 (55.17) | 35 (61.40) | 0.625 |  |
| Yogurt | 0 | 8 (13.79) | 3 (5.26) | 0.216 | 0.204 |
|  | 6 | 6 (10.34) | 4 (7.02) | 0.763 |  |
|  | 12 | 5 (8.62) | 5 (8.77) | 1.000 |  |
| Dairy | 0 | 9 (15.52) | 7 (12.28) | 0.817 | 0.179 |
|  | 6 | 8 (13.79) | 5 (8.77) | 0.578 |  |
|  | 12 | 9 (15.52) | 5 (8.77) | 0.412 |  |
| Grains | 0 | 28 (48.28) | 31 (54.39) | 0.639 | 0.471 |
|  | 6 | 31 (53.45) | 28 (49.12) | 0.781 |  |
|  | 12 | 27 (46.55) | 29 (50.88) | 0.781 |  |
| Vegetables | 0 | 31 (53.45) | 44 (77.19) | **0.013** | 0.610 |
|  | 6 | 35 (60.34) | 44 (77.19) | 0.081 |  |
|  | 12 | 29 (50.00) | 43 (75.44) | **0.009** |  |
| Fruits | 0 | 43 (74.14) | 44 (77.19) | 0.869 | 0.051 |
|  | 6 | 44 (75.86) | 40 (70.18) | 0.633 |  |
|  | 12 | 40 (68.97) | 40 (70.18) | 1.000 |  |
| Proteins  (meat, eggs, seafood) | 0 | 44 (75.86) | 46 (80.70) | 0.687 | 0.203 |
|  | 6 | 48 (82.76) | 44 (77.19) | 0.608 |  |
|  | 12 | 42 (72.41) | 44 (77.19) | 0.707 |  |
| Drinks | 0 | 8 (13.79) | 13 (22.81) | 0.313 | 0.549 |
|  | 6 | 6 (10.34) | 10 (17.54) | 0.398 |  |
|  | 12 | 6 (10.34) | 12 (21.05) | 0.186 |  |
| Snacks | 0 | 17 (29.31) | 15 (26.32) | 0.881 | 0.250 |
|  | 6 | 11 (18.97) | 15 (26.32) | 0.472 |  |
|  | 12 | 11 (18.97) | 13 (22.81) | 0.781 |  |
| Supplements | 0 | 9 (15.52) | 17 (29.82) | 0.107 | 0.499 |
|  | 6 | 12 (20.69) | 15 (26.32) | 0.623 |  |
|  | 12 | 8 (13.79) | 16 (28.07) | 0.098 |  |

^a^P value obtained via the chi-square test;

^b^P value obtained via a generalized linear mixed model.
